# Novel lncRNA-miRNA-mRNA Competing Endogenous RNA Triple Networks Associated Programmed Cell Death in Heart Failure

**DOI:** 10.3389/fcvm.2021.747449

**Published:** 2021-10-06

**Authors:** Yu Zheng, Yingjie Zhang, Xiu Zhang, Yini Dang, Yihui Cheng, Wenjie Hua, Meiling Teng, Shenrui Wang, Xiao Lu

**Affiliations:** ^1^Department of Rehabilitation Medicine, The First Affiliated Hospital of Nanjing Medical University, Nanjing, China; ^2^Department of Gastroenterology, The First Affiliated Hospital of Nanjing Medical University, Nanjing, China

**Keywords:** heart failure, competitive endogenous RNA, long non-coding RNA, programmed cell death, apoptosis, ferroptosis

## Abstract

**Objective:** Increasing evidence has uncovered the roles of lncRNA-miRNA-mRNA regulatory networks in cardiovascular diseases. However, the crosstalk between ceRNA networks and development of heart failure (HF) remains unclear. This study was to investigate the role of lncRNA-mediated ceRNA networks in the pathophysiological process of HF and its potential regulatory functions on programmed cell death.

**Methods:** We firstly screened the GSE77399, GSE52601 and GSE57338 datasets in the NCBI GEO database for screening differentially expressed lncRNAs, miRNAs and mRNAs. lncRNA-miRNA-mRNA regulatory networks based on the ceRNA theory were subsequently constructed. GO and KEGG enrichment analysis was conducted to predict potential biological functions of mRNAs in ceRNA networks. Differentially expressed mRNAs were then interacted with programmed cell death related genes. lncRNA-mediated ceRNA regulatory pathways on programmed cell death were validated with qRT-PCR testing.

**Results:** Based on our bioinformatic analysis, two lncRNAs, eight miRNAs and 65 mRNAs were extracted to construct two lncRNAs-mediated ceRNA networks in HF. Biological processes and pathways were enriched in extracellular matrix. Seven lncRNA-mediated ceRNA regulatory pathways on programmed cell death, GAS5/miR-345-5p/ADAMTS4, GAS5/miR-18b-5p/AQP3, GAS5/miR-18b-5p/SHISA3, GAS5/miR-18b-5p/C1orf105, GAS5/miR-18b-5p/PLIN2, GAS5/miR-185-5p/LPCAT3, and GAS5/miR-29b-3p/STAT3, were finally validated.

**Conclusions:** Two novel ceRNA regulatory networks in HF were discovered based on our bioinformatic analysis. Based on the interaction and validation analysis, seven lncRNA GAS5-mediated ceRNA regulatory pathways were hypothesized to impact programmed cell death including seven for apoptosis, three for ferroptosis, and one for pyroptosis. Upon which, we provided novel insights and potential research plots for bridging ceRNA regulatory networks and programmed cell death in HF.

## Introduction

Heart Failure (HF) is the terminal stage of various cardiovascular diseases. Advanced interventions, such as pharmacological treatment, cardiac resynchronous treatment, cardiac transplantation, mainly focus on HF related symptom control (1). With these interventions, the mortality of HF has been decreased to some extent (2). Nonetheless, novel therapeutic strategies underlying cellular and molecular pathways are warranted to further decrease the mortality and improve the quality of life in patients with HF. Accordingly, there is an imperative need for further research and deeper insight into the biological mechanisms underlying HF.

Non-coding RNAs (ncRNAs), such as microRNAs (miRNAs), long non-coding RNAs (lncRNAs) and circular RNAs (circRNAs), are a group of RNAs that play critical roles in cellular and molecular physiology and pathology including epigenetic, transcriptional regulation, and post-transcriptional regulation (3). Among these ncRNAs, increasing evidence indicates that lncRNA was widely involved in the pathological process of cardiac development, atherosclerosis, myocardial infarction, hypertension and aneurysm (4–6). Dysfunction of lncRNAs was also reported in a number of studies of HF (7, 8). Specifically, lncRNA MHRT and non-coding NFAT inhibitory factor NRON was found to significantly increase in the plasma of patients with HF (9). LncRNA LIPCA, functioning in the process of cardiac remodeling, was proved to be able to predict the long-term mortality in patients with chronic HF (10). However, evidence also suggests that lncRNAs may not perform modulatory functions independently while create dynamic regulatory crosstalk networks by interacting with other ncRNAs through competitively binding to certain ncRNAs, which was previously termed as the competitive endogenous RNA (ceRNA) network theory (11).

ceRNA network had been reported as a crucial mechanism to explain the regulation of post-transcriptional gene translation (12). It has been found that lncRNA APF targets the regulation of miR-188-3p, thereby affecting the expression of ATG7 in autophagy, which can effectively reduce the area of myocardial infarction, prevent HF, and prolong survival time (13). LncRNA kcnq1ot1 was demonstrated to increase HDAC3 expression by competitively binding to miR-452-3p, followed by the inhibition of ABCA1 and cholesterol efflflux, promoted macrophage lipid accumulation and accelerated the development of atherosclerosis (14). The above findings highlighted the importance of ceRNA network, a global view of lncRNA-mediated ceRNA network may help researchers comprehensively understand the pathophysiological process of cardiovascular diseases. Nonetheless, limited evidence was found in the research field of HF and more regulatory pathways still need further verification. Considering the essential role of programmed cell death in HF, it promoted us to think about the question that “whether there are interactions between lncRNA-mediated ceRNA network and programmed cell death?” Apart from one recent study of circRNA-miRNA-mRNA regulatory network on iron homeostasis (15), there were limited evidence to reveal the interactions between these two elements. The answers to this question may renew our knowledge in the regulation of pathophysiological process after HF.

Upon the above statement, this study was aimed to construct a global lncRNA-mediated triple network (lncRNA-miRNA-mRNA) based on the NCBI GEO dataset including lncRNA, miRNA and mRNA expression profiles. Differential gene expression profiles between HF subjects and healthy controls were analyzed with “Limma” package in R software, and lncRNA-miRNA-mRNA triple regulatory networks were constructed. Gene Ontology (GO) and Kyoto Encyclopedia of Genes and Genomes (KEGG) functional enrichment analyses for the differentially expressed mRNAs in the ceRNA network was analyzed. Afterwards, we interacted the differentially expressed mRNAs in the ceRNA network with genes related to programmed cell death in open source datasets. Finally, programmed cell death-related regulatory pathways in the lncRNA-mediated ceRNA network were further verified through quantitative real-time reverse transcription-polymerase chain reaction (qRT-PCR). This study may add insights into novel molecular mechanisms underlying HF pathogenesis, specifically interactions between lncRNA-mediated ceRNA regulatory networks and programmed cell death in the pathological process of HF, and finally provide novel treatment targets for patients with HF.

## Materials and Methods

**Figure 1:**
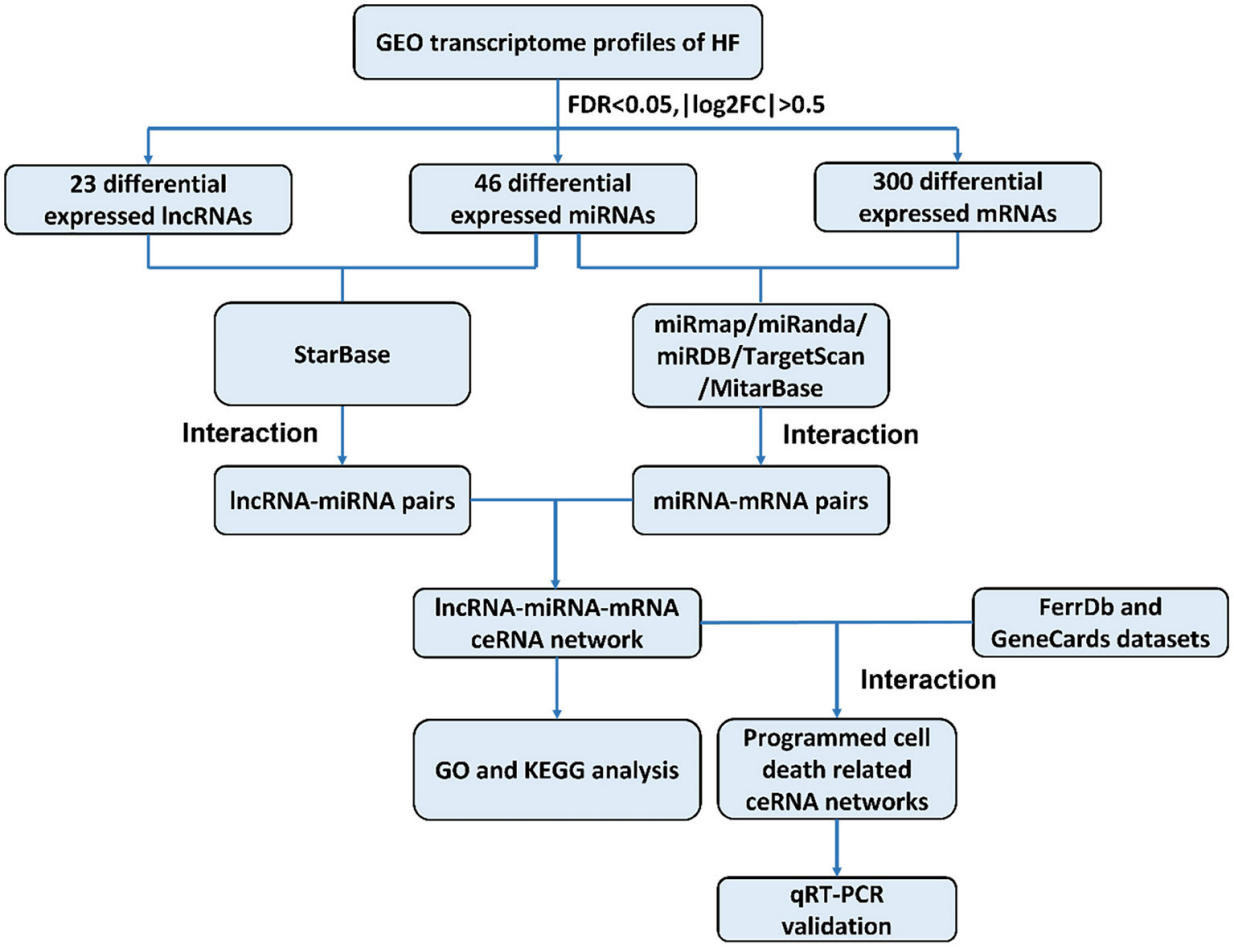
Flowchart of construction and analysis of ceRNA networks. GEO, gene expression omnibus; HF, heart failure; FDR, false discovery rate; FC, fold change; GO, Gene Ontology; KEGG, Kyoto Encyclopedia of Genes and Genomes; qRT-PCR, quantitative real-time reverse transcription-polymerase chain reaction.

The construction of a global lncRNA-mediated triple ceRNA network (lncRNA-miRNA-mRNA) was conducted following the logic shown in Figure 1.

### Online Data Collection

The gene expression profiles (GSE77399 for lncRNA, GSE52601 for miRNA and GSE57338 for mRNA) related to HF were downloaded from NCBI GEO (https://www.ncbi.nlm.nih.gov/geo/). The lncRNA data was collected with GPL21384 Platforms (Human Disease-related LncRNA Profiler, Molecular Cardiology, Milan, Italy), and included data from 13 patients with HF and 12 healthy controls. The miRNA array data were measured using GPL10558 Platforms (Illumina HumanHT-12 V4.0 expression beadchip, Laboratory of RNA Molecular Biology, Thomas Tuschl, New York, USA), in 16 patients with HF and eight healthy controls. The mRNA array data in GSE57338 was collected from 177 patients with HF and 136 healthy controls based on GPL11532 {Affymetrix Human Gene 1.1 ST Array [transcript (gene) version], Perelman School of Medicine at the University of Pennsylvania, Philadelphia, USA}.

### Screening Strategies for Differentially Expressed lncRNAs, miRNAs and mRNAs

Differential expression analysis of lncRNAs, miRNAs, and mRNAs between patients with HF and healthy controls was conducted with “Limma” package in the R software. The screening threshold for significant difference in gene expression was adjusted with *P* < 0.05 and |log2FC (fold change)|>0.5. For visualization, heat maps and volcano maps were generated by employing the “ggplot2” and “pheatmap” packages in the R software (16).

### Construction of the lncRNA-miRNA-mRNA Network

The lncRNA-miRNA-mRNA network was constructed based on ceRNA hypothesis as follows: (1) Interaction information of miRNA-mRNAs in the miRmap, miRanda, miRDB, TargetScan and MitarBase, and miRNA-lncRNAs in the StarBase were extracted (17–22); (2) if both the lncRNA and mRNA were targeted and were co-expressed negatively with one common miRNA, this lncRNA-miRNA-mRNA group was identified as co-expression competing triplet and the corresponding ceRNA regulatory networks were constructed. The ceRNA regulatory networks were visualized with Cytoscape 3.7.1 (23).

### GO and KEGG Functional Enrichment Analysis

Enriched GO terms (e.g., biological process, BP; cellular component, CC and molecular function, MF) and KEGG pathways were analyzed to predict potential biological functions of mRNAs underlying our ceRNA network with the “clusterprofiler” package in the R software (24). *P* < 0.05 was considered as statistically significant and results were visualized with bubble chart.

### Screening Strategy for Programmed Cell Death Related Genes in the ceRNA Network

We firstly interacted the differentially expressed mRNAs in our ceRNA network with genes related to programmed cell death in FerrDb (http://www.zhounan.org/ferrdb/) and GeneCards (https://www.genecards.org/). Afterwards, we interacted our differentially expressed mRNAs with genes related to apoptosis, pyroptosis and ferroptosis, respectively. Results were visualized by Venn diagram generated with the online tool Venny 2.1.0 [http://bioinfogp.cnb.csic.es/tools/venny/index.html (55)].

### Validation of Programmed Cell Death Related Genes in ceRNA Network in HF Animal Model

All the animal experimental protocols were in accordance with all institutional and national guideline for the care and use of laboratory animals and were reviewed and approved by the Ethics Committees of the Nanjing Medical University, Jiangsu Province, China. Healthy male Wistar rats were randomized either into the sham operation (SO) or the HF group. Under anesthesia, a thoracotomy was performed through the fourth intercostal space, the heart was exposed. A suture was placed 1-1.5 mm from the left anterior descending branch (LAD), and the ends were tied loosely in the SO group and firmly in the HF group (25). After 4 weeks, HF followed by myocardial infarction was confirmed by multiple morphological and hemodynamic parameters (mainly due to LVEF lower than 50%) and myocardial tissues were collected from the infarcted edge area of all eligible rats (26, 27).

For qRT-PCR testing of programmed cell death related ceRNA network, a template equivalent to 400 ng of total RNA extracted from myocardial tissues were subjected to 40 cycles of quantitative PCR using the Takara SYBR Premix Ex TaqTM on StepOnePlus™ Real-Time PCR System (Applied Biosystems, Foster City, California, USA). GAPDH and U6 were used as internal references and the relative expression level was calculated using the 2^−ΔΔCt^ method. Primer sequences used for two lncRNAs, seven miRNAs and 23 mRNAs were listed in Supplementary Table 1.

### Statistical Analysis

Data are expressed as the mean ± SD. Differentially expressed lncRNAs from the GSE77399 were compared with Student *t*-test. Differentially expressed miRNAs and mRNAs from the GSE52601 and the GSE57338, respectively, were compared with “Limma” package in R software. “Clusterprofiler” package in R software was used to perform GO and KEGG functional enrichment analysis for differentially expressed mRNAs in our ceRNA network. The results of qRT-PCT for validation were analyzed with Student *t*-test. *P* < 0.05 were considered significantly different. All analyses were performed using SPSS 25.0 (IBM, Chicago, Illinois, USA) and GraphPad Prism 8 (GraphPad Software, San Diego, California, USA).

## Results

### Identification of Differentially Expressed lncRNAs, miRNAs and mRNAs

**Figure 2:**
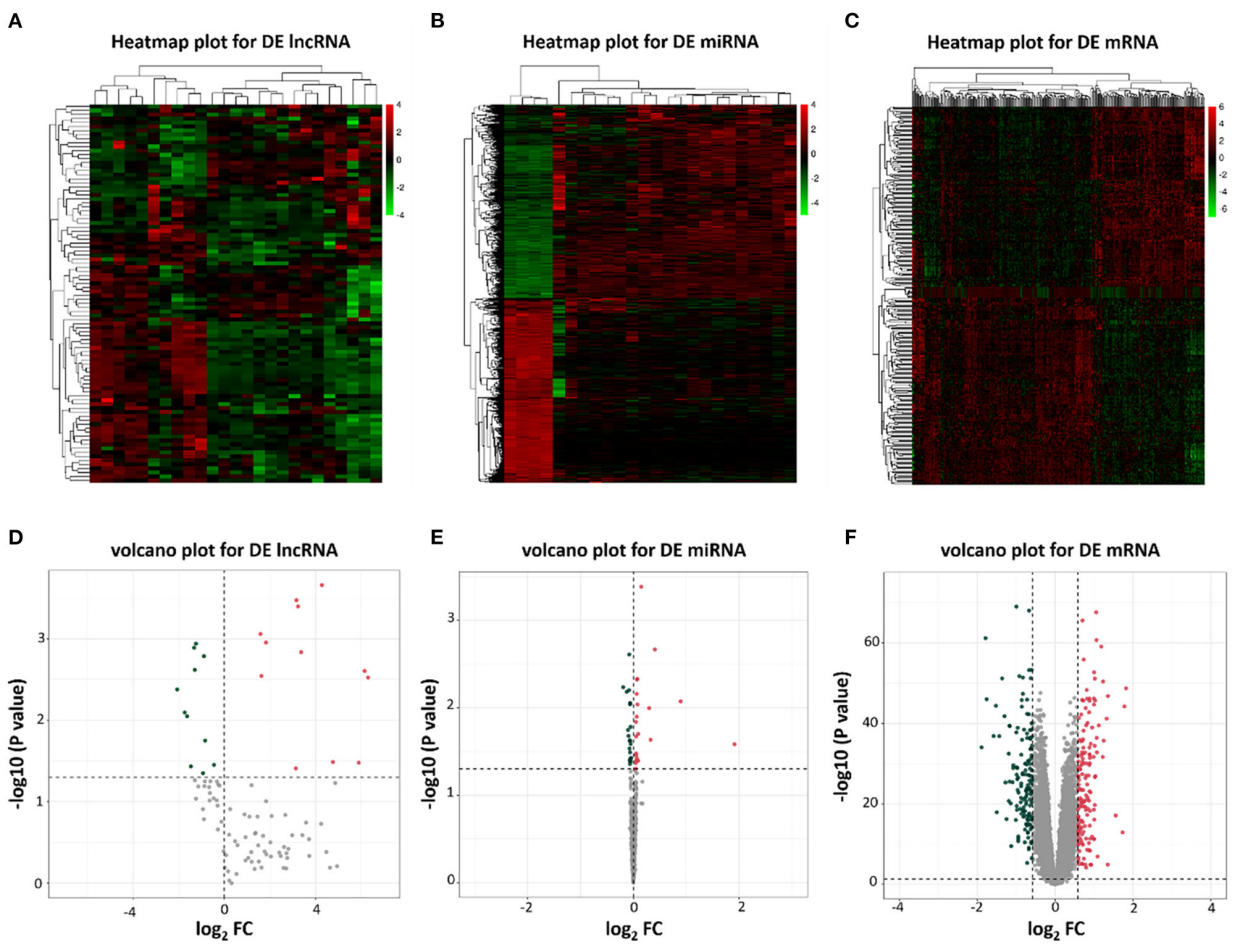
Heatmap plots and volcano plots of differentially expressed lncRNAs, miRNAs, and mRNAs. **(A)** Heatmap of differentially expressed lncRNAs; **(B)** Heatmap of differentially expressed miRNAs; **(C)** Heatmap of differentially expressed mRNAs; **(D)** Volcano plot of 23 differentially expressed lncRNAs; **(E)** Volcano plot of 46 differentially expressed miRNAs; **(F)** Volcano plot of 300 differentially expressed mRNAs. Red represents upregulated genes and green indicates downregulated genes. DE, differentially expressed; FC, fold change.

To identify potential differential lncRNAs, miRNAs and mRNAs in HF, a comparative analysis for expression profiles of lncRNAs, miRNAs and mRNAs between patients with HF and healthy controls using GEO dataset was performed with *P* < 0.05 and |log fold change [FC]|>0.5 as threshold. A total of 23 lncRNAs (12 upregulated and 11 downregulated), 46 miRNAs (22 upregulated and 24 downregulated) and 300 mRNAs (157 upregulated and 143 downregulated) were identified as differentially expressed genes between patients with HF and healthy controls (Supplementary Tables 2–4). The heatmap plots and volcano plots for differentially expressed lncRNAs, miRNAs and mRNAs were demonstrated in Figure 2.

### Construction of lncRNA–miRNA–mRNA ceRNA Network

**Figure 3:**
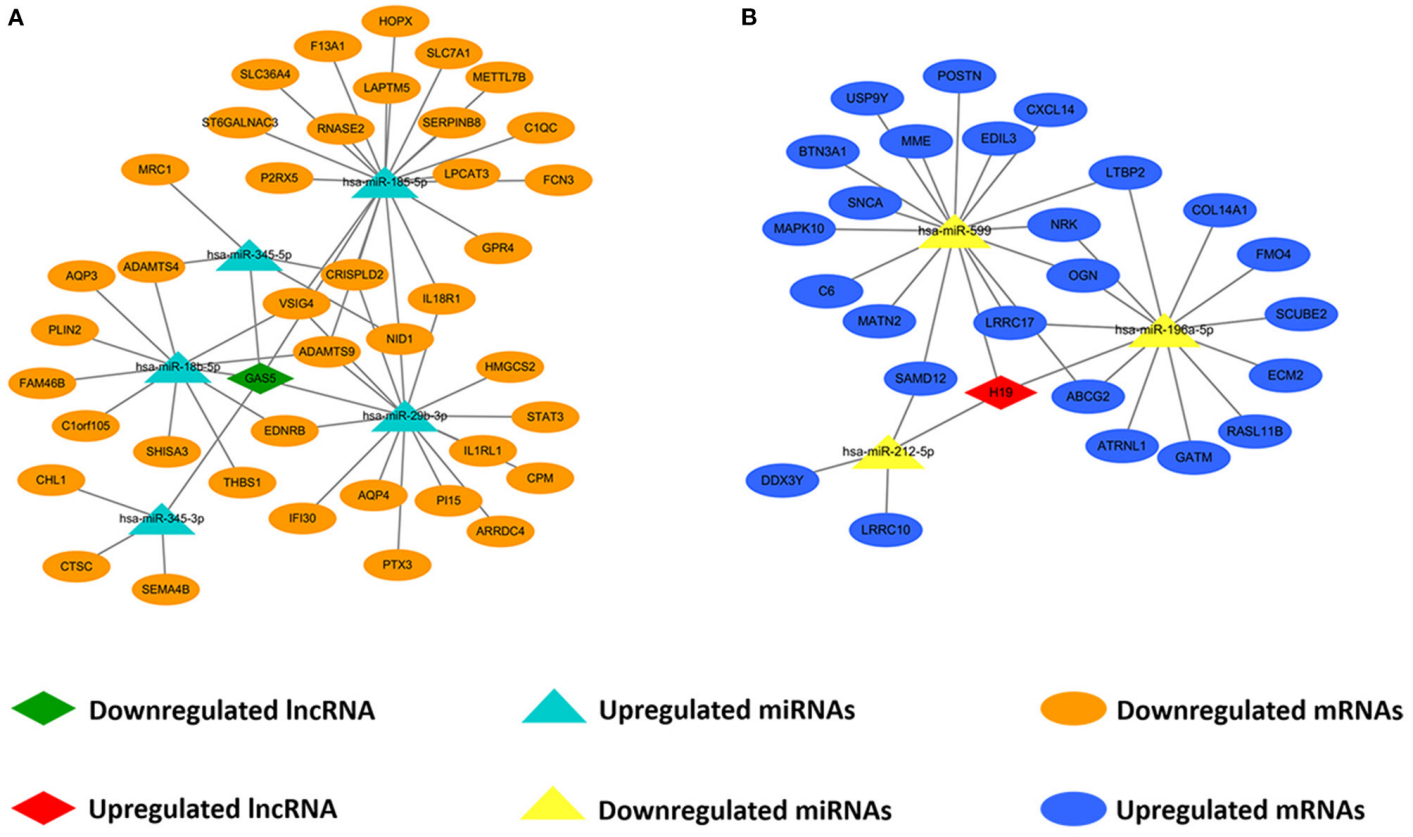
Construction of lncRNA-mediated ceRNA regulatory network. **(A)** The lncRNA-mediated downregulated ceRNA network; **(B)** The lncRNA-mediated upregulated ceRNA network. Green means downregulated LncRNA, red means upregulated LncRNA, blue indicates upregulated miRNAs and mRNAs, yellow represents downregulated miRNAs, orange represents downregulated mRNAs.

We predicted lncRNA-miRNA and miRNA-mRNA pairs according to both base sequence and expression level. Based on the interaction elements, five miRNA-lncRNA pairs and 51 miRNA-mRNA pairs were identified in the upregulated miRNA ceRNA network, and 3 miRNA-lncRNA pairs and 31 miRNA-mRNA pairs in the downregulated miRNA ceRNA network. Afterwards, the ceRNA regulatory networks were reconstructed, including one lncRNA node, five miRNA nodes and 40 mRNA nodes in the lncRNA mediated downregulated ceRNA network, and one lncRNA node, three miRNA nodes and 25 mRNA nodes in the lncRNA mediated upregulated ceRNA network (Figure 3).

### Functional Enrichment Analysis of Differentially Expressed mRNAs in ceRNA Networks

**Figure 4:**
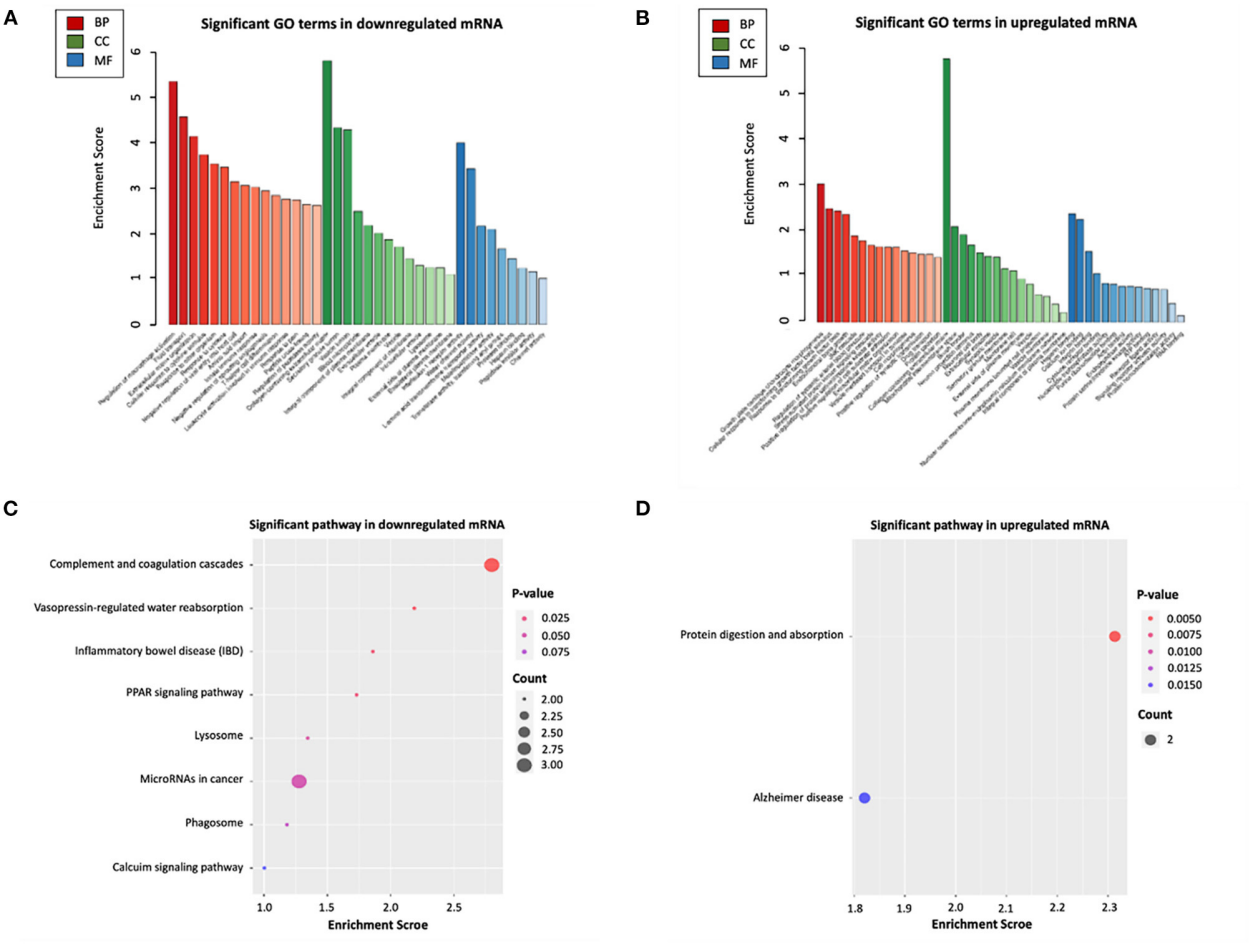
Functional enrichment analysis of differentially expressed mRNAs in ceRNA networks. **(A)** GO biological functional analyses of downregulated mRNA; **(B)** GO biological function analyses of upregulated mRNA; **(C)** KEGG pathway analyses of downregulated mRNA; **(D)** KEGG pathway analyses of upregulated mRNA. BP, biological process; CC, cellular component; MF, molecular function; GO, Gene Ontology.

To further explore the potential functions associated with our ceRNA network, functional enrichment analysis (including GO and KEGG) was utilized by “Clusterprofiler” package in R software. The results showed that the differentially expressed mRNAs participating in our ceRNA network were particularly enriched in the “extracellular matrix organization” (biological process), “collagen–containing extracellular matrix” (cellular component), “Interleukin−1 receptor activity” (molecular function), “regulation of systemic arterial blood pressure” (biological process), “mitochondrial intermembrane space” (cellular component), “RNA binding” (molecular function) interacted with upregulated or downregulated mRNA (Figures 4A,B). Additional KEGG pathway analysis indicated that differentially expressed mRNAs were relevant to “protein digestion and absorption,” “complement and coagulation cascades” and “vasopressin-regulated water reabsorption” in our ceRNA networks (Figures 4C,D).

### Identification of the Candidate Programmed Cell Death Related Genes in the ceRNA Networks

**Figure 5:**
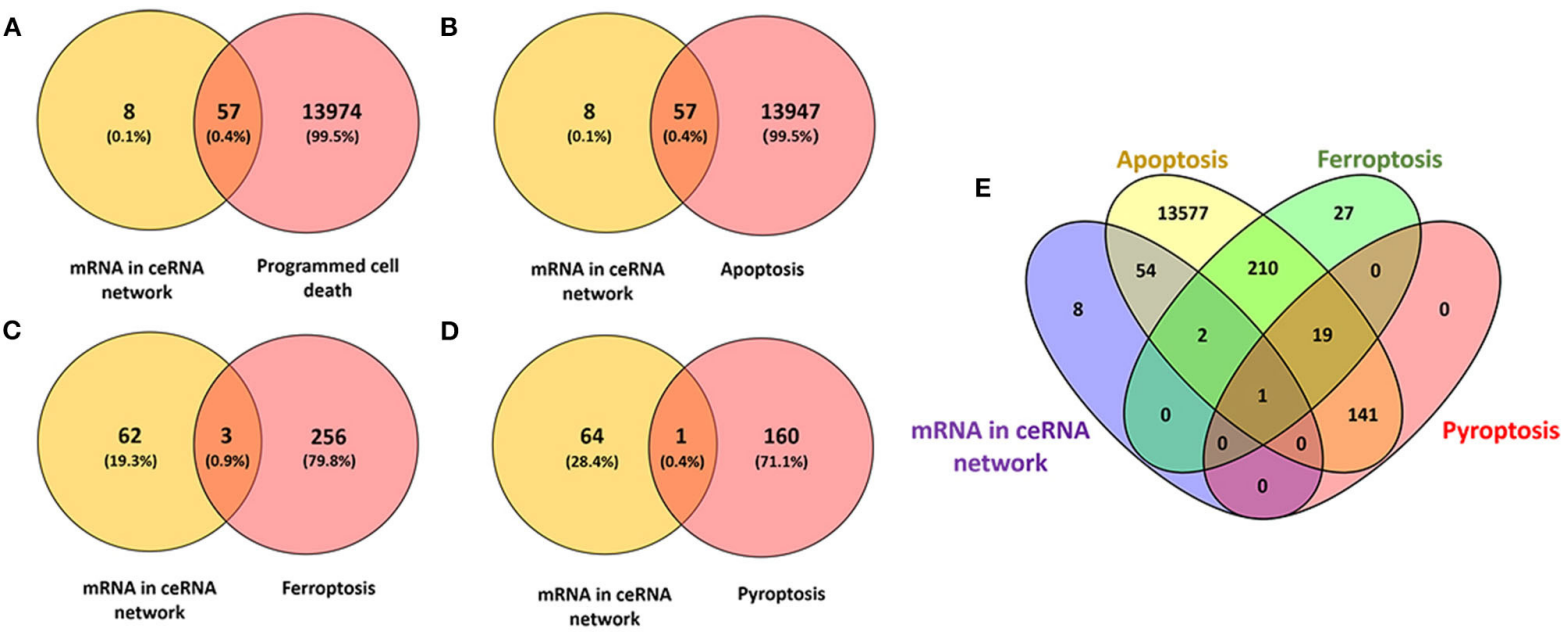
Identification of the candidate programmed cell death related genes in the ceRNA networks. **(A)** Venn diagram to identify common mRNAs between differentially expressed mRNAs in our ceRNA networks and programmed cell death related mRNAs; **(B)** Venn diagram to identify common mRNAs between differentially expressed mRNAs in our ceRNA networks and apoptosis related mRNAs; **(C)** Venn diagram to identify common mRNAs between differentially expressed mRNAs in our ceRNA networks and ferroptosis related mRNAs; **(D)** Venn diagram to identify common mRNAs between differentially expressed mRNAs in our ceRNA networks and pyroptosis related mRNAs; **(E)** Interactions between differentially expressed mRNAs in our ceRNA networks and apoptosis, ferroptosis, pyroptosis related mRNAs.

We interacted 65 differentially expressed mRNAs in our ceRNA networks with 14,031 programmed cell death related genes in FerrDb and GeneCards, 57 common genes were consequently obtained (Figure 5A; Supplementary Table 5). Afterwards, 65 differentially expressed mRNAs in our ceRNA networks were interacted with 14,004 apoptosis related genes in GeneCards, 57 common genes were obtained (Figure 5B; Supplementary Table 5). With regard to ferroptosis, three common genes in our ceRNA networks were identified (Figure 5C; Supplementary Table 5) and one pyroptosis related common gene was identified (Figure 5D; Supplementary Table 5).

### Validation of Genes in the Programmed Cell Death Regulatory Pathways

**Figure 6:**
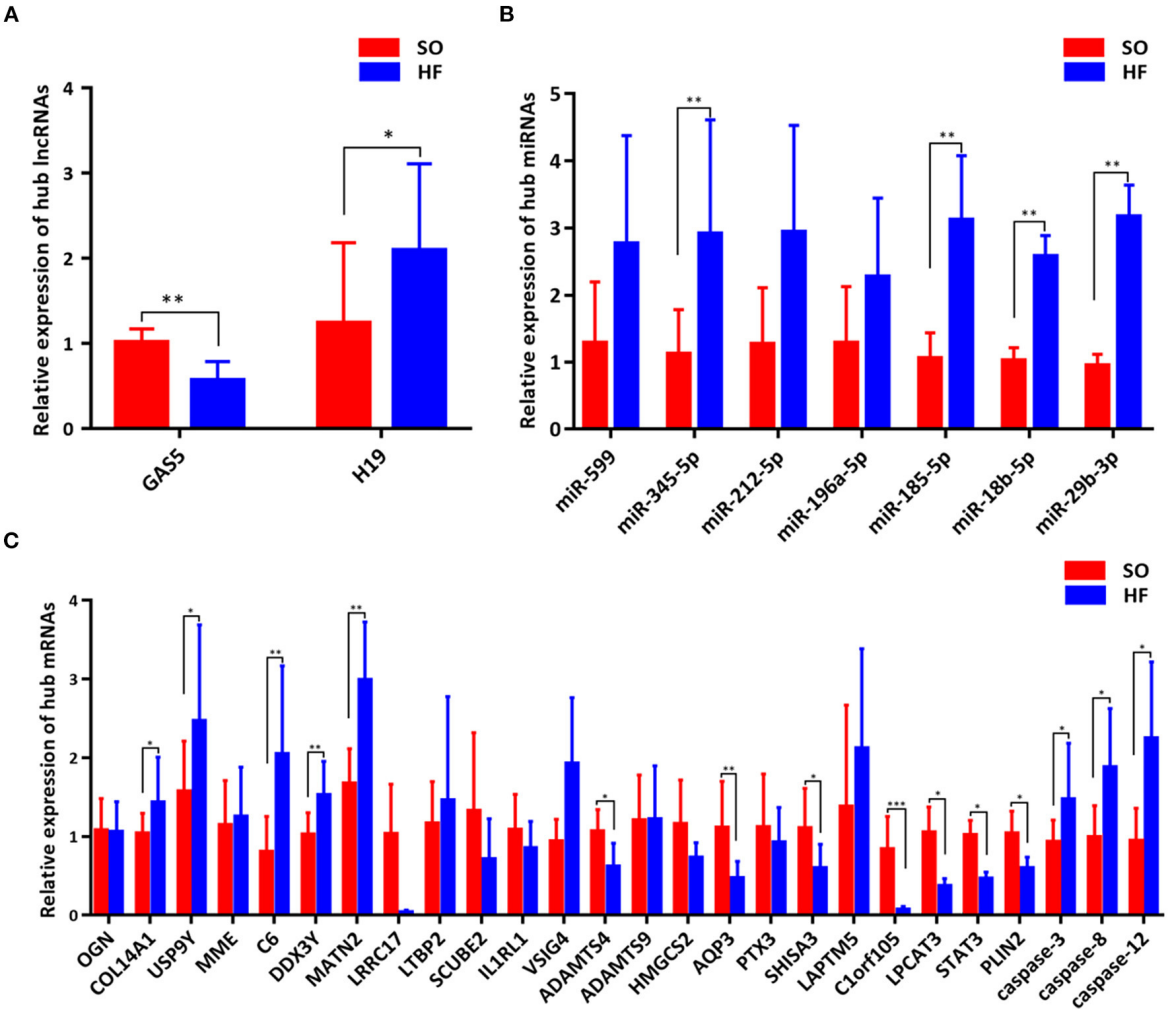
Validation of genes in the programmed cell death regulatory pathways. **(A)** Expression of hub lncRNAs in ceRNA networks; **(B)** Expression of hub miRNAs in ceRNA networks; **(C)** Expression of hub mRNAs in ceRNA networks. *P < 0.05; **P < 0.01; ***P < 0.001.

To validate the expression of programmed cell death related genes, a number of lncRNAs, miRNAs, and mRNAs were selected for qRT-PCR testing. Specifically, two lncRNAs in the ceRNA networks were selected. Twenty apoptosis related genes including top 10 upregulated and downregulated mRNAs in our ceRNA networks (Figure 6C) and three common apoptosis related genes (caspase-3, caspase-8 and caspase-12, Figure 6C), three ferroptosis related mRNAs (LPCAT3, STAT3 and PLIN2, Figure 6C), and one pyroptosis related gene (STAT3, Figure 6C) were included. Finally, the above mRNAs targeted miRNAs were also selected for qRT-PCR validation.

**Figure 7:**
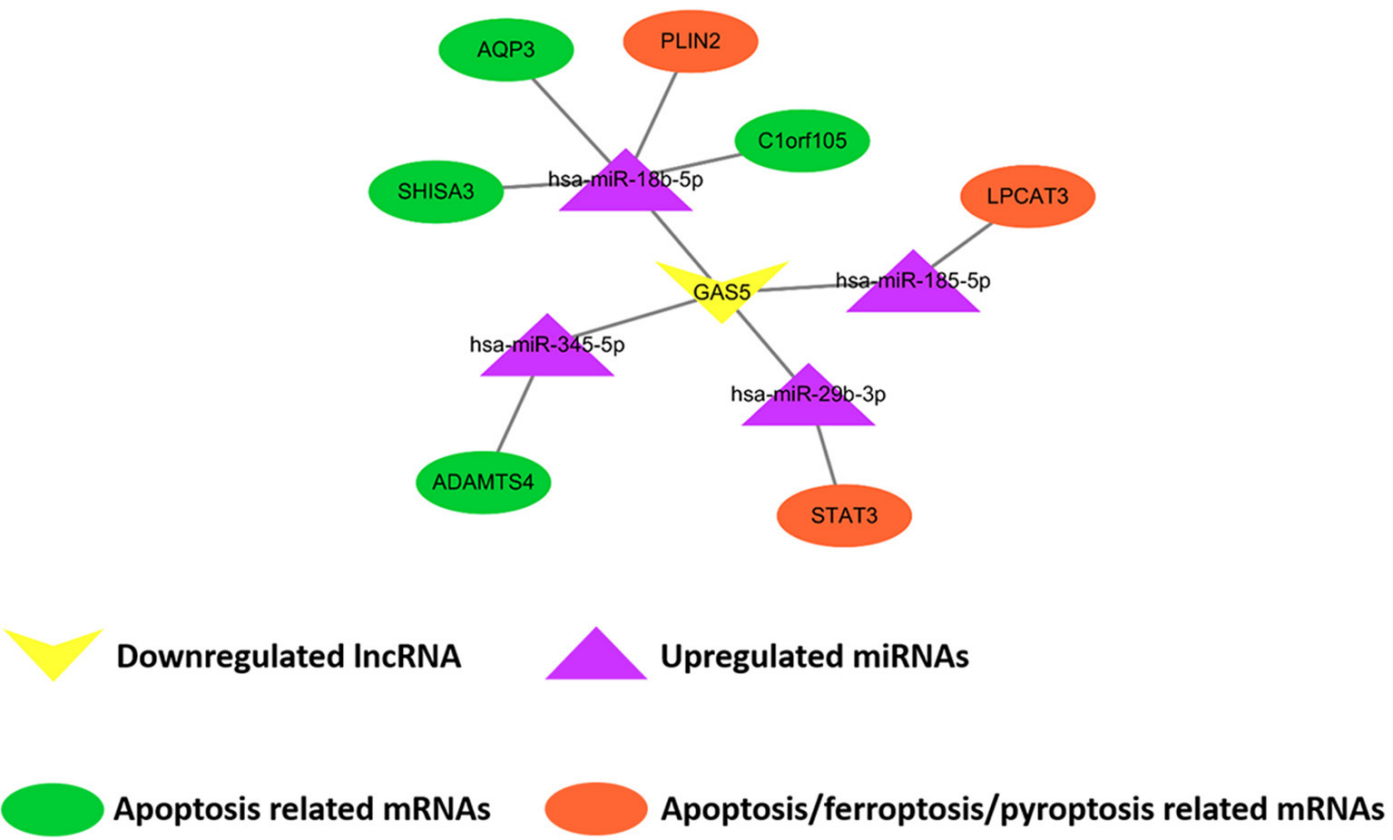
Demonstration of proposed lncRNA GAS5-mediated ceRNA regulatory networks on programmed cell death. The rectangle, triangles, and circles represent lncRNAs, miRNAs and mRNAs, respectively. The circles highlighted in orange and green indicate apoptosis related mRNAs, the circles highlighted in orange indicate ferroptosis related mRNAs, and the circle highlighted in orange (STAT3) indicates pyroptosis related mRNAs.

Comparing SO with HF, qRT-PCR results showed that lncRNA GAS5 was significantly upregulated while lncRNA H19 was significantly downregulated (Figure 6A). miR-345-5p, miR-185-5p, miR-18b-5p, miR-29b-3p were significantly upregulated in HF (Figure 6B). In addition, ferroptosis related genes, LPCAT3, STAT3, and PLIN2, were confirmed to be differentially downregulated in HF by qRT-PCR analyses (Figure 6C). For apoptosis related genes, COL14A1, USP9Y, C6, DDX3Y and MATN2 were significantly upregulated (Figure 6C) while ADAMTS4, AQP3, SHISA3, and C1orf105 were significantly downregulated in HF (Figure 6C). Finally, we proposed seven lncRNA GAS5-mediated ceRNA regulatory pathways on programmed cell death, and the results are presented in Figure 7; Supplementary Table 6.

## Discussion

In the present study, we demonstrated the lncRNA-mediated ceRNA regulatory networks in HF. We screened the NCBI GEO database and constructed two lncRNA-miRNA-mRNA ceRNA regulatory networks based on the ceRNA theory, including two lncRNAs, eight miRNAs and 65 mRNAs. GO and KEGG enrichment analysis demonstrated that differentially expressed mRNAs participating in our ceRNA network were particularly enriched in the “extracellular matrix organization” and “collagen–containing extracellular matrix.” 57 lncRNA-mediated ceRNA regulatory pathways were detected after the interaction between our ceRNA networks with programmed cell death related genes. Finally, qRT-PCR testing demonstrated seven ceRNA regulatory pathways to be significantly associated with programmed cell death in the pathophysiological process of HF, including lncRNA GAS5/miR-345-5p/ADAMTS4, lncRNA GAS5/miR-18b-5p/AQP3, lncRNA GAS5/miR-18b-5p/SHISA3, lncRNA GAS5/miR-18b-5p/C1orf105, lncRNA GAS5/miR-18b-5p/PLIN2, lncRNA GAS5/miR-185-5p/LPCAT3, and lncRNA GAS5/miR-29b-3p/STAT3.

Two ceRNA networks nested with two hub nodes (lncRNAs), including downregulated lncRNA GAS5 and upregulated lncRNA H19, were detected in our comprehensive bioinformatic analysis. lncRNA GAS5, as a growth arrest specific transcription factor, is capable to regulate cell growth, survival and proliferation (28). Recent evidence suggested that the expression of lncRNA GAS5 in chronic HF was downregulated, while the expression of miR-223-3p was upregulated. lncRNA GAS5 and miR-223-3p was demonstrated to predict the occurrence and recurrence of CHF based on the ROC curve analysis (29). In addition, the expression of lncRNA H19 and its encoded miR-675 were verified to be up-regulated in pathological cardiac hypertrophy and HF (30). lncRNA H19 was reported to be upregulated in decompensated right ventricular, further silencing H19 limited pathological right ventricular hypertrophy, fibrosis and capillary rarefaction. However, the dynamic regulatory crosstalk between these lncRNAs with other transcripts is unknown. Consequently, our newly discovered lncRNA-mediated ceRNA regulatory networks may provide novel insights in understanding the mechanisms underlying the initiation and progression of HF.

To further explore the biological functions of differentially expressed mRNAs in HF, we performed GO and KEGG pathway analysis of 65 differentially expressed mRNAs in the ceRNA network. We found these mRNAs were significantly enriched in the “extracellular matrix organization” and “collagen–containing extracellular matrix.” This is consistent with previous points of view that cardiac remodeling is accompanied by several cellular changes, such as cardiomyocyte hypertrophy, myocyte apoptosis and necrosis, fibroblast proliferation, accumulation of proinflammatory mediators, and extracellular matrix reorganization characterized by fibrosis induction (31). Tanshinone IIA, traditional Chinese medicine to protect against organ injuries, reversed the increased expression of collagen I, collagen III, MMP-2 and MMP-9 in HF rat model and improved cardiac dysfunction and fibrosis (32). An observational, prospective, longitudinal study of outpatients with HF found collagen turnover biomarkers (e.g., MMP-2) combined with clinical, biochemical and echocardiographic characteristics can improve the predictive precision of cardiovascular prognosis at the time of diagnosis (33). In addition, mRNAs in our ceRNA networks were significantly enriched in the “complement and coagulation cascade.” In patients with stable systolic HF, increased C3c levels were associated with less adverse cardiac remodeling and improved survival rate (34). Therefore, it was reasonable to hypothesize that our newly constructed ceRNA networks may play a role in the previously reported dysregulated alternative pathway of complement activation in patients with chronic HF. The combined effect of alternative pathways mediated by the ceRNA network in inflammation and fibrogenesis may be the underlying mechanisms of the development of diastolic dysfunction (35).

Apart from the above biological functions extracted from GO and KEGG pathway analysis, it is believed that featured programmed cell death is essential in the regulation of cardiomyocyte death in HF (36–38). Programmed cell death represents the primary means through which the organism coordinates the elimination of damaged cells at risk of neoplastic transformation or those hijacked by microbes for pathogen replication, including apoptosis, ferroptosis, and pyroptosis (38). Here, we interacted the significantly differentially expressed mRNAs in our ceRNA networks with genes related to programmed cell death. In our networks, we obtained 57 mRNAs related to these three sub-types of programmed cell death. Seven ceRNA regulatory pathways were finally validated with qRT-PCR testing.

Among the above ceRNA regulatory pathways, several hub nodes have been demonstrated to play crucial roles in cardiovascular diseases. For example, ADAMTS4, known as a secreted proteinase involved in inflammation and matrix degradation, was reported to translocated to the nucleus in smooth muscle cell (SMCs), through cleaving and degrading poly ADP ribose polymerase-1, leading to SMC apoptosis (39). STAT3 signaling was demonstrated to be activated by IL-35, followed by inhibited cytochrome C release and reduced apoptosis signaling, and finally protected cardiomyocytes against mtROS-induced apoptosis (40). Celastrol, an anti-inflammatory and anti-apoptotic agent, could antagonize high glucose-induced cardiomyocyte apoptosis and inflammation through restraining miR-345-5p (41). Overexpression or knockdown of miR-29b-3p showed its crucial roles on regulation of apoptosis and production of pro-inflammatory cytokines in rat cardiac myocytes (42). However, the impact of dynamic regulatory crosstalk networks involving the above hub nodes on these downstream functional changes in HF has not been well-studied. Our newly detected ceRNA networks were very likely to exert regulatory functions not only for apoptosis but potentially for all sub-types of programmed cell death, and in turn impact the pathophysiological process of HF. Even though, the detailed mechanisms for each step need further validation.

The other sub-type of programmed cell death, ferroptosis, has gained attention recently (43–45). It is an iron-dependent form of regulated cell death that is characterized by the accumulation of lipid hydroperoxides to lethal levels, resulting in oxidative damage to cell membranes. Recent studies have preliminarily uncovered the links between ferroptosis and cardiovascular diseases (46). Previous studies found that ferroptosis was involved in HF, however few studies in depth explored the regulatory mechanisms of ferroptosis in HF (47, 48). In our analysis, GAS5/miR-18b-5p/PLIN2, GAS5/miR-185-5p/LPCAT3, and GAS5/miR-29b-3p/STAT3 were potentially linked to ferroptosis. Unfortunately, the ferroptosis related hub nodes detected in our study were only explored in non-HF diseases. For example, RNA-seq analysis indicated PLIN2 was an indispensable gene in the suppression of ferroptosis caused by abnormal lipometabolism in gastric carcinoma (49). Another study demonstrated that LPCAT3 triggered the process of Arachidonoyl (AA)-CoA converted to AA-phosphatidylethanolamine (PE), the latter promoted esterification and ultimately led to ferroptosis (43). In addition, phosphorylated STAT3 could upregulate the expression of SLC7A11 and reduce ferroptosis, thereby improving the pathological processes associated with acute lung injury (50). As the most focused mRNA, while considering the crosstalk between ceRNA network mediated STAT3 regulation and ferroptosis, evidence was only reported in cancers but not in HF (51–54). Nonetheless, our promising results provided potential research spots and information for linking ferroptosis and ceRNA regulatory networks in HF, the functional changes and specific interactions between ferroptosis and ceRNA regulatory networks are warranted to be further explored. With the clarification of the role lncRNA GAS5 plays in the pathophysiological process of HF, it may serve as biomedical target for the treatment of HF. In addition, clinical measurement of lncRNA GAS5 may benefit diagnostic process and contribute to prognostic prediction of HF.

This study also has several limitations. Firstly, our study was conducted according to genetic information downloaded from GEO database while sequencing analysis (e.g., DNA-seq, RNA-seq) of samples obtained from human or animal is recommended. Nonetheless, enlarged human cohort validating the observations or well-designed animal studies exploring the comprehensive mechanisms cannot be ruled out. Secondly, our hypothesized potential binding affinity between lncRNAs, miRNAs, and mRNAs should be further experimentally investigated. Last but not the least, should any of our newly proposed seven lncRNA GAS5-mediated ceRNA regulatory networks on programmed cell death to be validated, it would step forward the clinical diagnosis and treatment of HF to some extent.

## Conclusions

In conclusion, we newly found two ceRNA regulatory networks in HF including two lncRNAs, eight miRNAs and 65 mRNAs based on the bioinformatic analysis. Their potential target mRNAs were validated and suggested to mainly impact extracellular matrix. Apart from the biological functions extracted from GO and KEGG enrichment analysis, seven newly discovered lncRNA GAS5-mediated ceRNA regulatory pathways, were hypothesized and validated to impact programmed cell death including seven for apoptosis (lncRNA GAS5/miR-345-5p/ADAMTS4, lncRNA GAS5/miR-18b-5p/AQP3, lncRNA GAS5/miR-18b-5p/SHISA3, lncRNA GAS5/miR-18b-5p/C1orf105, lncRNA GAS5/miR-18b-5p/PLIN2, lncRNA GAS5/miR-185-5p/LPCAT3, and lncRNA GAS5/miR-29b-3p/STAT3), three for ferroptosis (lncRNA GAS5/miR-18b-5p/PLIN2, lncRNA GAS5/miR-185-5p/LPCAT3, and lncRNA GAS5/miR-29b-3p/STAT3), and one for pyroptosis (lncRNA GAS5/miR-29b-3p/STAT3). Based on these results, we provided novel insights and potential research plots for bridging programmed cell death and ceRNA regulatory networks in HF.

## Data Availability Statement

The datasets presented in this study can be found in online repositories. The names of the repository/repositories and accession number(s) can be found in the article Supplementary Material.

## Ethics Statement

The animal study was reviewed and approved by the Ethics Committees of the Nanjing Medical University, Jiangsu Province, China.

## Author Contributions

XL, YZhe, and YD conceived of and designed the research plans. XZ and YZha completed data processing and bioinformatics analyses. YZha, XZ, YC, WH, MT, SW, and XZ established the HF rat model and YZha isolated RNA samples from myocardial tissues and performed qRT-PCR testing. YZhe, XZ, and YZha drafted the manuscript with contributions from all the authors. YZhe and YD supervised and modified the drafting process. All authors contributed to the article and approved the submitted version.

## Funding

This study was funded by the National Natural Science Foundation of China (Grant number: 81772441, 81902288, and 82072546).

## Conflict of Interest

The authors declare that the research was conducted in the absence of any commercial or financial relationships that could be construed as a potential conflict of interest.

## Publisher's Note

All claims expressed in this article are solely those of the authors and do not necessarily represent those of their affiliated organizations, or those of the publisher, the editors and the reviewers. Any product that may be evaluated in this article, or claim that may be made by its manufacturer, is not guaranteed or endorsed by the publisher.
