# Supplementary material for: Novel lncRNA-miRNA-mRNA Competing Endogenous RNA Triple Networks Associated Programmed Cell Death in Heart Failure
Source: Front Cardiovasc Med. 2021 Oct 6;8:747449. doi: 10.3389/fcvm.2021.747449 (PMC8528160; doi:10.3389/fcvm.2021.747449)
Supplement: Supplementary file 1 [file Table_1.DOCX]

**Supplementary Tables 1-6**

**Table S1 Primer sequences of RNAs for qRT-PCR**

| **Gene Name** | **Species** | **Sequence** |
| --- | --- | --- |
| GAPDH | Rat | F:5' GCATCTTCTTGTGCAGTGCC 3'  R:5' ACCAGCTTCCCATTCTCAGC 3' |
| U6 | Rat | F:5' CTCGCTTCGGCAGCACA 3'  R:5' AACGCTTCACGAATTTGCGT 3' |
| GAS5 | Rat | F:5' GGTGAGTCTGAGGTGTGCAT 3'  R:5' TGAAACCGTGGATTTGGCCT 3' |
| H19 | Rat | F:5' CATGACATGGTCCGGTGTGA 3' |
|  |  | R:5' CCAGACATGAGCTGGGTAGC 3' |
| miR-599 | Rat | F:5' ACACTCCAGCTGGGGTTGTGTCAGTTTA 3' |
|  |  | R:5' CTCAACTGGTGTCGTGGAGTCGGCAATTCAGTTGAGGTTTGATA 3' |
| miR-345-5p | Rat | F:5' ACACTCCAGCTGGGTGCTGACCCCTAGTC 3' |
|  |  | R:5' CTCAACTGGTGTCGTGGAGTCGGCAATTCAGTTGAGGCACTGGA 3' |
| miR-212-5p | Rat | F:5' ACACTCCAGCTGGGACCTTGGCTCTAGACTGC 3' |
|  |  | R:5' CTCAACTGGTGTCGTGGAGTCGGCAATTCAGTTGAGCAGTAAGC 3' |
| miR-196a-5p | Rat | F:5' ACACTCCAGCTGGGTAGGTAGTTTCATGTT 3' |
|  |  | R:5' CTCAACTGGTGTCGTGGAGTCGGCAATTCAGTTGAGCCCAACAA 3' |
| has-miR-29b-3p | Rat | F:5' ACACTCCAGCTGGGTAGCACCATTTGAAATC 3'  R:5' CTCAACTGGTGTCGTGGAGTCGGCAATTCAGTTGAGAACACTGA 3' |
| has-miR-185-5p | Rat | F:5' ACACTCCAGCTGGGTGGAGAGAAAGGCAGT 3'  R:5' CTCAACTGGTGTCGTGGAGTCGGCAATTCAGTTGAGTCAGGAAC 3' |
| has-miR-18b-5p | Rat | F:5' ACACTCCAGCTGGGTAAGGTGCATCTAGTGC 3'  R:5' CTCAACTGGTGTCGTGGAGTCGGCAATTCAGTTGAG CTAACTGC 3' |
| OGN | Rat | F:5' AGATGCTCCAGAACCTGTGC 3' |
|  |  | R:5' CATGTGGCTCCCCTTTCCTT 3' |
| COL14A1 | Rat | F:5' GGAGAGGGACAAGGAGAGGA 3' |
|  |  | R:5' CAACGTTCCAGCCCAGGTT 3' |
| USP9Y | Rat | F:5' ACCACAAGAAACTCATGAAAGCA 3' |
|  |  | R:5' GCAGGTTGTGTATATAACCACAGTA 3' |
| MME | Rat | F:5' ACACAAACTCTGGGGTGAGC 3' |
|  |  | R:5' ACCTGAAGAACAAGGACGCA 3' |
| C6 | Rat | F:5' AGATCGCTTTAAAGGGCCTGG 3' |
|  |  | R:5' ACAGAGCTGATCGCATGAGT 3' |
| DDX3Y | Rat | F:5' AAGATGAGCTTGGGCTGGAC 3' |
|  |  | R:5' ACTTGGCTCTCGTGTCACTG 3' |
| MATN2 | Rat | F:5' CCTTTGTGTGAACAGTGGCG 3' |
|  |  | R:5' GGGCCTTCAGTGCATCTCTT 3' |
| LRRC17 | Rat | F:5' GCTGCAGGACAGAGAACACT 3' |
|  |  | R:5' CCCACCCAGGAAGATGGAAC 3' |
| LTBP2 | Rat | F:5' GGTCATCCCTGAGGAGGAGT 3' |
|  |  | R:5' GGGTCCTAGATGGAGGTGGT 3' |
| SCUBE2 | Rat | F:5' CTGGATGACTGCCATGCTGA 3' |
|  |  | R:5' AGCGATAGTTGCCTGGGATG 3' |
| IL1RL1 | Rat | F:5' AGAGGAAGAAGTGCCTGGGA 3' |
|  |  | R:5' CCCAAAGCCCCATTCTCCAT 3' |
| VSIG4 | Rat | F:5' CCTATGGCCATCCCGTCCTA 3' |
|  |  | R:5' GGCCTCTGTATTTTGCCTGC 3' |
| ADAMTS4 | Rat | F:5' CAATGGAGATCCGGAGTCGG 3' |
|  |  | R:5' CCAGAAGGAGCCTTGACGTT 3' |
| ADAMTS9 | Rat | F:5' ACAGGGCCGTTTTAGCATCA 3' |
|  |  | R:5' CCTTCAGTACAGAGCCACCG 3' |
| HMGCS2 | Rat | F:5' TGTGTCAGATCTGCCCAAACG 3' |
|  |  | R:5' GGGGAATGGTTGTATGGATTGG 3' |
| AQP3 | Rat | F:5' CAGCTCGTGACTTTGGACCT 3' |
|  |  | R:5' GGGCCAGCTTCACATTCTCT 3' |
| PTX3 | Rat | F:5' TATGGCACCAAGTGGAACCC 3' |
|  |  | R:5' TGGCCATCTCCAGAGTGGTA 3' |
| SHISA3 | Rat | F:5' CCTGGCAATCATGGCGAATG 3' |
|  |  | R:5' ACTGGCCCTTCGAGTTTACG 3' |
| LAPTM5 | Rat | F:5' AGCCCTGGCCATCTACCATA 3' |
|  |  | R:5' CGGTTCTTGACCACTCCGAA 3' |
| C1orf105 | Rat | F:5' GGGAGTTTGAAGGCAGACTTG 3' |
|  |  | R:5' GGGCGACTGGTGATATGGAG 3' |
| STAT3 | Rat | F:5' CTAACCGGATCGCTGAGGTACA 3'  R:5' GGAGCTGGTTCCACTGAGCC 3' |
| LPCAT3 | Rat | F:5' TGAGCCTTAACAAGTTGGCG 3'  R:5' GGTAGAACTGGTGGCCGAAG 3' |
| PLIN2 | Rat | F:5' TCGTCTCTCAGCTCTCCTGT 3'  R:5' CTGTCACAGCCACTGAGGTC 3' |

**Table S2 Identification of differentially expressed lncRNA in HF**

|  | **Upregulated** | | |  |  | **Downregulated** |  |
| --- | --- | --- | --- | --- | --- | --- | --- |
| **IncRNA** | | **logFC** | **P value** | | **IncRNAs** | **logFC** | **P value** |
| RMRP | | 6.299 | 0.003 | | LINCRNA-SFMBT2 | -2.068 | 0.004 |
| H19 | | 6.149 | 0.002 | | LINCRNA-VLDLR | -1.737 | 0.008 |
| Y4 | | 5.891 | 0.033 | | ROR | -1.630 | 0.009 |
| HOXA11AS | | 4.759 | 0.033 | | PR-AT2 | -1.468 | 0.037 |
| ANRIL | | 4.278 | 0.000 | | PCAT-1 | -1.322 | 0.001 |
| LUST | | 3.366 | 0.001 | | HOTAIR | -1.294 | 0.002 |
| SOX2OT | | 3.230 | <0.001 | | GAS5 | -1.241 | 0.001 |
| KRASP1 | | 3.160 | <0.001 | | PCAT-43 | -0.935 | 0.045 |
| BC017743 | | 3.135 | 0.039 | | LOC285194 | -0.893 | 0.002 |
| BC043430 | | 1.826 | 0.001 | | NDM29 | -0.841 | 0.018 |
| BACE1AS | | 1.625 | 0.003 | | MER11C | -0.453 | 0.035 |
| SRA | | 1.585 | 0.001 | |  |  |  |

HF: heart failure; FC: fold change.

**Table S3 Identification of differentially expressed miRNA in HF**

|  | **Upregulated** |  |  | **Downregulated** |  |
| --- | --- | --- | --- | --- | --- |
| **miRNA** | **logFC** | **P value** | **miRNA** | **logFC** | **P value** |
| miR1974 | 1.913 | 0.026 | miR1228 | -0.193 | 0.006 |
| miR1978 | 0.895 | 0.008 | miR586 | -0.126 | 0.007 |
| miR1185-1 | 0.407 | 0.002 | miRLET7D | -0.102 | 0.018 |
| miR129-2 | 0.325 | 0.023 | miR607 | -0.091 | 0.021 |
| miR345 | 0.299 | 0.010 | miR551B | -0.081 | 0.006 |
| miR29B1 | 0.151 | <0.001 | miR1323 | -0.079 | 0.002 |
| miR635 | 0.092 | 0.040 | miR942 | -0.072 | 0.029 |
| miR185 | 0.092 | 0.020 | miR489 | -0.069 | 0.024 |
| miR611 | 0.078 | 0.009 | miR555 | -0.068 | 0.040 |
| miR1202 | 0.074 | 0.005 | miR365-2 | -0.067 | 0.041 |
| miR920 | 0.065 | 0.005 | miR212 | -0.067 | 0.023 |
| miR18B | 0.063 | 0.038 | miR503 | -0.065 | 0.044 |
| miR1266 | 0.062 | 0.013 | miR599 | -0.063 | 0.032 |
| miR449A | 0.062 | 0.007 | miR196A1 | -0.063 | 0.009 |
| miR376A1 | 0.060 | 0.040 | miR1256 | -0.063 | 0.009 |
| miR450A2 | 0.055 | 0.040 | miR374A | -0.062 | 0.041 |
| miR874 | 0.051 | 0.033 | miR328 | -0.061 | 0.009 |
| miR483 | 0.051 | 0.021 | miR499 | -0.060 | 0.024 |
| miR124-2 | 0.048 | 0.035 | miR154 | -0.053 | 0.017 |
| miR146B | 0.048 | 0.050 | miR15B | -0.053 | 0.038 |
| miR337 | 0.047 | 0.014 | miR939 | -0.051 | 0.026 |
| miR554 | 0.046 | 0.043 | miR199B | -0.051 | 0.041 |
|  |  |  | miR570 | -0.050 | 0.039 |
|  |  |  | miR92B | -0.043 | 0.037 |

HF: heart failure; FC: fold change.

**Table S4 Identification of differentially expressed mRNA (Top 30) in HF**

|  | **Upregulated** |  |  | **Downregulated** |  |
| --- | --- | --- | --- | --- | --- |
| **mRNA** | **logFC** | **P value** | **mRNA** | **logFC** | **P value** |
| ASPN | 1.823 | <0.001 | SERPINA3 | -2.639 | <0.001 |
| AFRP4 | 1.777 | <0.001 | PLA2G2A | -1.890 | <0.001 |
| NPPA | 1.733 | <0.001 | FCN3 | -1.787 | <0.001 |
| HBB | 1.555 | <0.001 | IL1RL1 | -1.759 | <0.001 |
| FRZB | 1.357 | <0.001 | MYH6 | -1.579 | <0.001 |
| EIE1AY | 1.351 | <0.001 | CD163 | -1.520 | <0.001 |
| OGN | 1.322 | <0.001 | SERPINE1 | -1.497 | <0.001 |
| COL14A1 | 1.246 | <0.001 | LYVE1 | -1.435 | <0.001 |
| LUM | 1.231 | <0.001 | SLCO4A1 | -1.361 | <0.001 |
| MXRA5 | 1.223 | <0.001 | VSIG4 | -1.299 | <0.001 |
| SMOC2 | 1.186 | <0.001 | CYP4B1 | -1.275 | <0.001 |
| IFI44L | 1.121 | <0.001 | AREG | -1.248 | <0.001 |
| USP9Y | 1.089 | <0.001 | ADAMTS4 | -1.191 | <0.001 |
| CCRL1 | 1.071 | <0.001 | MIR208A | -1.189 | <0.001 |
| PHLDA1 | 1.066 | <0.001 | ADAMTS9 | -1.172 | <0.001 |
| MNS1 | 1.062 | <0.001 | AOX1 | -1.170 | <0.001 |
| FREM1 | 1.055 | <0.001 | RNASE2 | -1.164 | <0.001 |
| SFRP1 | 1.035 | <0.001 | MGST1 | -1.140 | <0.001 |
| FNDC1 | 1.027 | <0.001 | HMGCS2 | -1.127 | <0.001 |
| P116 | 1.022 | <0.001 | ANKRD2 | -1.093 | <0.001 |
| PDE5A | 1.019 | <0.001 | METTL7B | -1.075 | <0.001 |
| HAPLN1 | 1.017 | <0.001 | MYOT | -1.072 | <0.001 |
| MME | 1.015 | <0.001 | S100A8 | -1.009 | <0.001 |
| C6 | 1.005 | <0.001 | TUBA3D | -0.995 | <0.001 |
| ECM2 | 1.000 | <0.001 | C1QTNF1 | -0.994 | <0.001 |
| HBA2 | 0.999 | <0.001 | ANPEP | -0.992 | <0.001 |
| HBA1 | 0.999 | <0.001 | FCER1G | -0.987 | <0.001 |
| PTN | 0.998 | <0.001 | SGPP2 | -0.985 | <0.001 |
| NRK | 0.993 | <0.001 | PI15 | -0.981 | <0.001 |
| DSC1 | 0.993 | <0.001 | FKBP5 | -0.971 | <0.001 |

HF: heart failure; FC: fold change.

**Table S5 Identified lncRNA-mediated ceRNAs and the intersection between programmed cell death related genes and mRNA in the ceRNA networks**

| **lncRNA** | **miRNA** | **mRNA** | **Programmed cell death** | **Apoptosis** | **Ferroptosis** | **Pyroptosis** |
| --- | --- | --- | --- | --- | --- | --- |
| GAS5 | hsa-miR-345-5p | MRC1 | X | X |  |  |
| GAS5 | hsa-miR-345-5p | CRISPLD2 | X | X |  |  |
| GAS5 | hsa-miR-345-5p | NID1 |  |  |  |  |
| GAS5 | hsa-miR-345-5p | ADAMTS4 | X | X |  |  |
| GAS5 | hsa-miR-345-3p | CHL1 | X | X |  |  |
| GAS5 | hsa-miR-345-3p | CTSC | X | X |  |  |
| GAS5 | hsa-miR-345-3p | SEMA4B | X | X |  |  |
| GAS5 | hsa-miR-18b-5p | AQP3 | X | X |  |  |
| GAS5 | hsa-miR-18b-5p | FAM46B |  |  |  |  |
| GAS5 | hsa-miR-18b-5p | ADAMTS9 | X | X |  |  |
| GAS5 | hsa-miR-18b-5p | THBS1 | X | X |  |  |
| GAS5 | hsa-miR-18b-5p | SHISA3 | X | X |  |  |
| GAS5 | hsa-miR-18b-5p | ADAMTS4 | X | X |  |  |
| GAS5 | hsa-miR-18b-5p | C1orf105 | X | X |  |  |
| GAS5 | hsa-miR-18b-5p | PLIN2 | X | X | X |  |
| GAS5 | hsa-miR-18b-5p | EDNRB | X | X |  |  |
| GAS5 | hsa-miR-18b-5p | VSIG4 | X | X |  |  |
| GAS5 | hsa-miR-185-5p | SLC36A4 |  |  |  |  |
| GAS5 | hsa-miR-185-5p | VSIG4 | X | X |  |  |
| GAS5 | hsa-miR-185-5p | C1QC | X | X |  |  |
| GAS5 | hsa-miR-185-5p | METTL7B |  |  |  |  |
| GAS5 | hsa-miR-185-5p | IL18R1 | X | X |  |  |
| GAS5 | hsa-miR-185-5p | FCN3 | X | X |  |  |
| GAS5 | hsa-miR-185-5p | P2RX5 | X | X |  |  |
| GAS5 | hsa-miR-185-5p | NID1 |  |  |  |  |
| GAS5 | hsa-miR-185-5p | F13A1 | X | X |  |  |
| GAS5 | hsa-miR-185-5p | RNASE2 |  |  |  |  |
| GAS5 | hsa-miR-185-5p | SLC7A1 | X | X |  |  |
| GAS5 | hsa-miR-185-5p | HOPX | X | X |  |  |
| GAS5 | hsa-miR-185-5p | LPCAT3 | X | X | X |  |
| GAS5 | hsa-miR-185-5p | LAPTM5 | X | X |  |  |
| GAS5 | hsa-miR-185-5p | ADAMTS9 | X | X |  |  |
| GAS5 | hsa-miR-185-5p | SERPINB8 | X | X |  |  |
| GAS5 | hsa-miR-185-5p | GPR4 | X | X |  |  |
| GAS5 | hsa-miR-185-5p | ST6GALNAC3 |  |  |  |  |
| GAS5 | hsa-miR-185-5p | CRISPLD2 |  |  |  |  |
| GAS5 | hsa-miR-29b-3p | ARRDC4 | X | X |  |  |
| GAS5 | hsa-miR-29b-3p | HMGCS2 | X | X |  |  |
| GAS5 | hsa-miR-29b-3p | NID1 |  |  |  |  |
| GAS5 | hsa-miR-29b-3p | IFI30 | X | X |  |  |
| GAS5 | hsa-miR-29b-3p | ADAMTS9 | X | X |  |  |
| GAS5 | hsa-miR-29b-3p | PI15 |  |  |  |  |
| GAS5 | hsa-miR-29b-3p | AQP4 | X | X |  |  |
| GAS5 | hsa-miR-29b-3p | PTX3 | X | X |  |  |
| GAS5 | hsa-miR-29b-3p | IL1RL1 | X | X |  |  |
| GAS5 | hsa-miR-29b-3p | CRISPLD2 | X | X |  |  |
| GAS5 | hsa-miR-29b-3p | STAT3 | X | X | X | X |
| GAS5 | hsa-miR-29b-3p | VSIG4 | X | X |  |  |
| GAS5 | hsa-miR-29b-3p | IL18R1 | X | X |  |  |
| GAS5 | hsa-miR-29b-3p | CPM | X | X |  |  |
| GAS5 | hsa-miR-29b-3p | EDNRB |  |  |  |  |
| H19 | hsa-miR-196a-5p | FMO4 | X | X |  |  |
| H19 | hsa-miR-196a-5p | ECM2 |  |  |  |  |
| H19 | hsa-miR-196a-5p | RASL11B | X | X |  |  |
| H19 | hsa-miR-196a-5p | ABCG2 | X | X |  |  |
| H19 | hsa-miR-196a-5p | SCUBE2 | X | X |  |  |
| H19 | hsa-miR-196a-5p | NRK | X | X |  |  |
| H19 | hsa-miR-196a-5p | ATRNL1 |  |  |  |  |
| H19 | hsa-miR-196a-5p | COL14A1 | X | X |  |  |
| H19 | hsa-miR-196a-5p | LTBP2 | X | X |  |  |
| H19 | hsa-miR-196a-5p | OGN | X | X |  |  |
| H19 | hsa-miR-196a-5p | GATM | X | X |  |  |
| H19 | hsa-miR-196a-5p | LRRC17 | X | X |  |  |
| H19 | hsa-miR-212-5p | SAMD12 | X | X |  |  |
| H19 | hsa-miR-212-5p | LRRC10 | X | X |  |  |
| H19 | hsa-miR-212-5p | DDX3Y | X | X |  |  |
| H19 | hsa-miR-599 | NRK | X | X |  |  |
| H19 | hsa-miR-599 | USP9Y | X | X |  |  |
| H19 | hsa-miR-599 | LTBP2 | X | X |  |  |
| H19 | hsa-miR-599 | SNCA | X | X |  |  |
| H19 | hsa-miR-599 | C6 | X | X |  |  |
| H19 | hsa-miR-599 | ABCG2 | X | X |  |  |
| H19 | hsa-miR-599 | BTN3A1 | X | X |  |  |
| H19 | hsa-miR-599 | MATN2 | X | X |  |  |
| H19 | hsa-miR-599 | OGN | X | X |  |  |
| H19 | hsa-miR-599 | POSTN | X | X |  |  |
| H19 | hsa-miR-599 | MME | X | X |  |  |
| H19 | hsa-miR-599 | LRRC17 | X | X |  |  |
| H19 | hsa-miR-599 | CXCL14 | X | X |  |  |
| H19 | hsa-miR-599 | SAMD12 | X | X |  |  |
| H19 | hsa-miR-599 | MAPK10 | X | X |  |  |
| H19 | hsa-miR-599 | EDIL3 | X | X |  |  |

**Table S6 Proposed seven lncRNA GAS5-mediated programmed cell death related ceRNA regulatory networks**

| **lncRNA** | **miRNA** | **mRNA** | **Programmed cell death** | **Apoptosis** | **Ferroptosis** | **Pyroptosis** |
| --- | --- | --- | --- | --- | --- | --- |
| GAS5 | hsa-miR-345-5p | ADAMTS4 | X | X |  |  |
| GAS5 | hsa-miR-18b-5p | AQP3 | X | X |  |  |
| GAS5 | hsa-miR-18b-5p | SHISA3 | X | X |  |  |
| GAS5 | hsa-miR-18b-5p | C1orf105 | X | X |  |  |
| GAS5 | hsa-miR-18b-5p | PLIN2 | X | X | X |  |
| GAS5 | hsa-miR-185-5p | LPCAT3 | X | X | X |  |
| GAS5 | hsa-miR-29b-3p | STAT3 | X | X | X | X |
